# Large slip, long duration, and moderate shaking of the Nicaragua 1992 tsunami earthquake caused by low near-trench rock rigidity

**DOI:** 10.1126/sciadv.abg8659

**Published:** 2021-08-06

**Authors:** Valentí Sallarès, Manel Prada, Sebastián Riquelme, Adrià Meléndez, Alcinoe Calahorrano, Ingo Grevemeyer, César R. Ranero

**Affiliations:** 1 Institute of Marine Sciences (ICM), CSIC, Barcelona, Spain.; 2 National Seismological Center, University of Chile, Santiago, Chile.; 3 GEOMAR Helmholtz Centre for Ocean Research, Kiel, Germany.; 4 Institució Catalana de Recerca i Estudis Avançats (ICREA), Barcelona, Spain.

## Abstract

Large earthquake ruptures propagating up to areas close to subduction trenches are infrequent, but when they occur, they heavily displace the ocean seafloor originating destructive tsunamis. The current paradigm is that the large seafloor deformation is caused by local factors reducing friction and increasing megathrust fault slip, or prompting the activation of ancillary faults or energy sources. As alternative to site-specific models, it has been proposed that large shallow slip could result from depth-dependent rock rigidity variations. To confront both hypotheses, here, we map elastic rock properties across the rupture zone of the *M*_S_7.0-*M*_W_7.7 1992 Nicaragua tsunami earthquake to estimate a property-compatible finite fault solution. The obtained self-consistent model accounts for trenchward increasing slip, constrains stress drop, and explains key tsunami earthquake characteristics such as long duration, high-frequency depletion, and magnitude discrepancy. The confirmation that these characteristics are all intrinsic attributes of shallow rupture opens new possibilities to improve tsunami hazard assessment.

## Teaser

Elastic rock properties in the Nicaragua subduction zone explain the anomalous characteristics of tsunami earthquakes.

## INTRODUCTION

### Near-trench earthquake rupture and seafloor deformation

Modeling of devastating tsunamis such as those originated by the giant *M*_W_9.1 2004 Sumatra-Andaman (*1*) and *M*_W_9.0 2010 Tohoku-Oki (*2*) megathrust earthquakes, the two largest ones of this century, reveals the occurrence of an extraordinarily large near-trench sea-bottom displacement. This is the case not only of the largest megathrust earthquakes but also of smaller events such as “tsunami earthquakes,” which generate disproportionately large tsunamis for their surface wave magnitude (*3*). However, the origin and underlying causes of the inferred seafloor deformation are still disputed. The dominant hypothesis for both giant and tsunami earthquakes is that deformation results from a large slip along the shallow portion of the megathrust fault. While there is direct evidence that rupture reached the trench in some of these events [e.g., Tohoku Oki (*4*, *5*)], the occurrence of near-trench slip is difficult to reconcile with the common assumption, based on frictional rock properties, that the shallowest 5 to 10 km of the megathrust should behave aseismically (*6*). To solve this issue, it has been suggested that fault friction might be locally low (*7*) as a result of site-specific factors and conditions like the presence of weak subducting sediments (*8*), fluid overpressure (*9*), and low shear stresses (*10*) or resulting from dynamic weakening (*11*). None of these processes, however, entails trenchward increasing slip by themselves. It has also been proposed that the vertical component of the seafloor displacement could be either generated or amplified by the presence of local features such as splay faults (*12*) and subducting seamounts (*13*) or by variations of slab dip (*14*). Last, some tsunamis have been suggested to be boosted by ancillary energy sources such as landslides (*15*), by inelastic folding (*16*) or horizontal displacement (*17*) of the seafloor, or by the release of gravitational potential energy (*18*). What all these mechanisms, which have been proposed to explain source properties of both tsunami earthquakes and giant earthquakes rupturing to near the trench, have in common is that they are not general but site and earthquake dependent.

As alternative to site-specific features and conditions, the global model of Sallarès and Ranero (SR) proposes that diverse anomalous source properties of shallow earthquake ruptures, including the presumed large slip, could arise from systematic variations of upper plate rock rigidity with depth (*19*). If this were the case, then measuring the elastic rock properties of any subduction zone would suffice to predict first-order earthquake source characteristics as a function of rupture depth. The SR model naturally predicts an increasing slip when rupture approaches the trench, and in addition, it explains the overall trends of longer duration, pronounced high-frequency depletion, and large moment magnitude (*M*_W_)–surface wave magnitude (*M*_S_) difference that are common to tsunami earthquakes (*3*, *20*, *21*) and to near-trench rupture portions of large megathrust earthquakes (*22*–*25*) and smaller shallow events (*26*, *27*). However, the validity of the SR model has yet to be tested for any individual event rupturing the shallowest megathrust segment. Here, we do so for the particular case of the 1992 Nicaragua tsunami earthquake. We first map the elastic rock properties across the rupture zone of this event, and we use them to estimate an elastic property–consistent moment release distribution and to infer other source characteristics that are then contrasted with observations. This is a particularly relevant comparison because those earthquake and tsunami have previously been associated to weak subducting sediment (*28*), submarine landslides (*29*), and subducting seamounts (*30*) or to subduction erosion along the plate boundary (*31*), similar to other shallow events.

### The 1992 Nicaragua tsunami earthquake

**Fig. 1:**
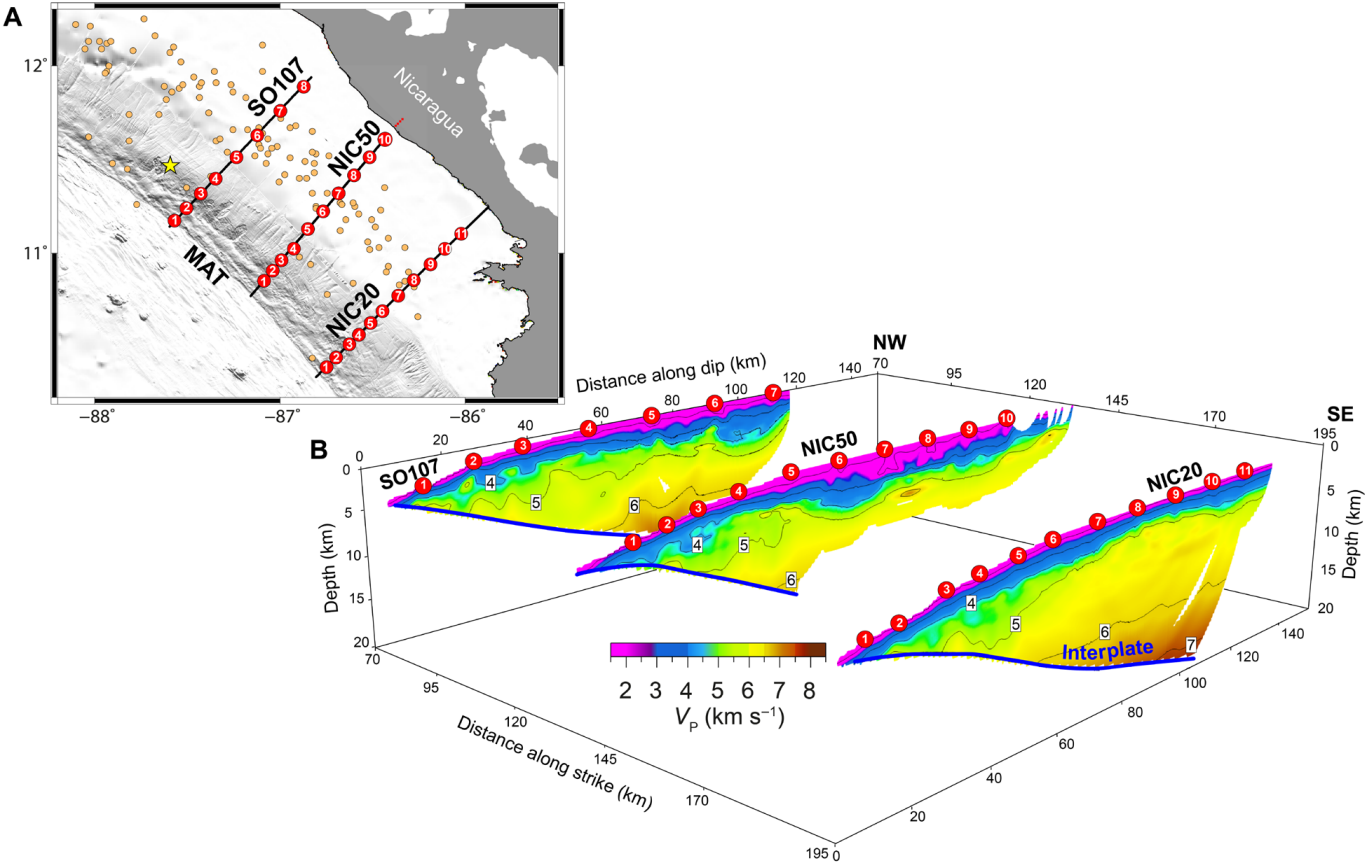
Map of the study zone and *P*-wave velocity models. (**A**) Seafloor relief map of the study area including the WAS and MCS profiles used in this study (i.e., NIC80/SO107, NIC50, and NIC20). Red circles indicate ocean bottom hydrophone (OBH) locations, while red squares are land stations, and the yellow star is the epicenter location of the *M*_W_7.6 to 7.7, 2 September 1992 Nicaragua earthquake (*34*). Orange circles are aftershocks. MAT, Middle America Trench. (**B**) 3D view of the 2D *P*-wave velocity (*V*_P_) models obtained by joint refraction and reflection travel-time tomography along each profile in (A). Colors indicate *V*_P_ according to the color scale. The blue thick line follows the inverted interplate boundary. We masked tomographic models with their corresponding ray coverage to show only those regions of the model resolved by the inversion.

On 2 September 1992, a large tsunami with average run-up heights of 3 to 8 m, locally reaching near 10 m, swept the Nicaraguan coasts (*32*–*35*), leaving 170 people dead, almost 500 injured, and more than 13,500 homeless. Although the epicenter was located just ~120 km off the coast (Fig. 1A), ground motion was mild, reaching a maximum intensity of III in the modified Mercalli scale (*36*), so it was hardly felt at some coastal areas and the tsunami hit the coast unexpectedly (*37*). *M*_S_ was 7.0 to 7.2, too small for the tsunami size, whereas *M*_W_ was 7.6 to 7.7, so that the *M*_W_*-M*_S_ difference was up to 0.7, anomalously large for the earthquake’s magnitude (*28*, *32*). The event nucleated at a depth of 10 to 15 km (*33*, *34*), but the moment release and the largest slip triggering the tsunami appear to have concentrated shallower than 10 km (*32*, *36*, *38*–*41*). The source duration was about 100 to 150 s, anomalously long for the rupture surface, indicating a slow rupture propagation (*38*, *42*–*44*). The source of low-frequency radiation concentrated in the zone of largest moment release (*33*), originating the high-frequency depletion of the released seismic moment, which caused, in turn, the moderate ground shaking (*21*).

In the case of the Nicaragua 1992 tsunami earthquake, published models matching seismological data and tsunami heights (*33*, *36*–*38*, *43*–*45*) include sundry simplifications, assumptions, and ad hoc values of rock properties that are not based on field data or observations. In this work, we use *P*-wave seismic velocity (*V*_P_) and interplate geometry obtained along three seismic profiles to derive the two-dimensional (2D) distribution of upper plate elastic rock properties throughout the rupture area of this event (Fig. 1). We then estimate a moment release and slip distribution by finite fault inversion, imposing a depth-varying rigidity extracted from the 2D model, which is then used to constrain stress drop variations throughout the earthquake’s rupture zone. This approach allows us to obtain a self-consistent solution that reproduces a number of characteristic features of this tsunami earthquake, including rupture duration, moment spectrum, and *M*_W_*-M*_S_ discrepancy, and to identify, in turn, source properties and seismic record attributes that are intrinsic to shallow ruptures with strong tsunamigenic potential.

## RESULTS

### Elastic rock properties across the rupture area of the 1992 tsunami earthquake

**Fig. 2:**
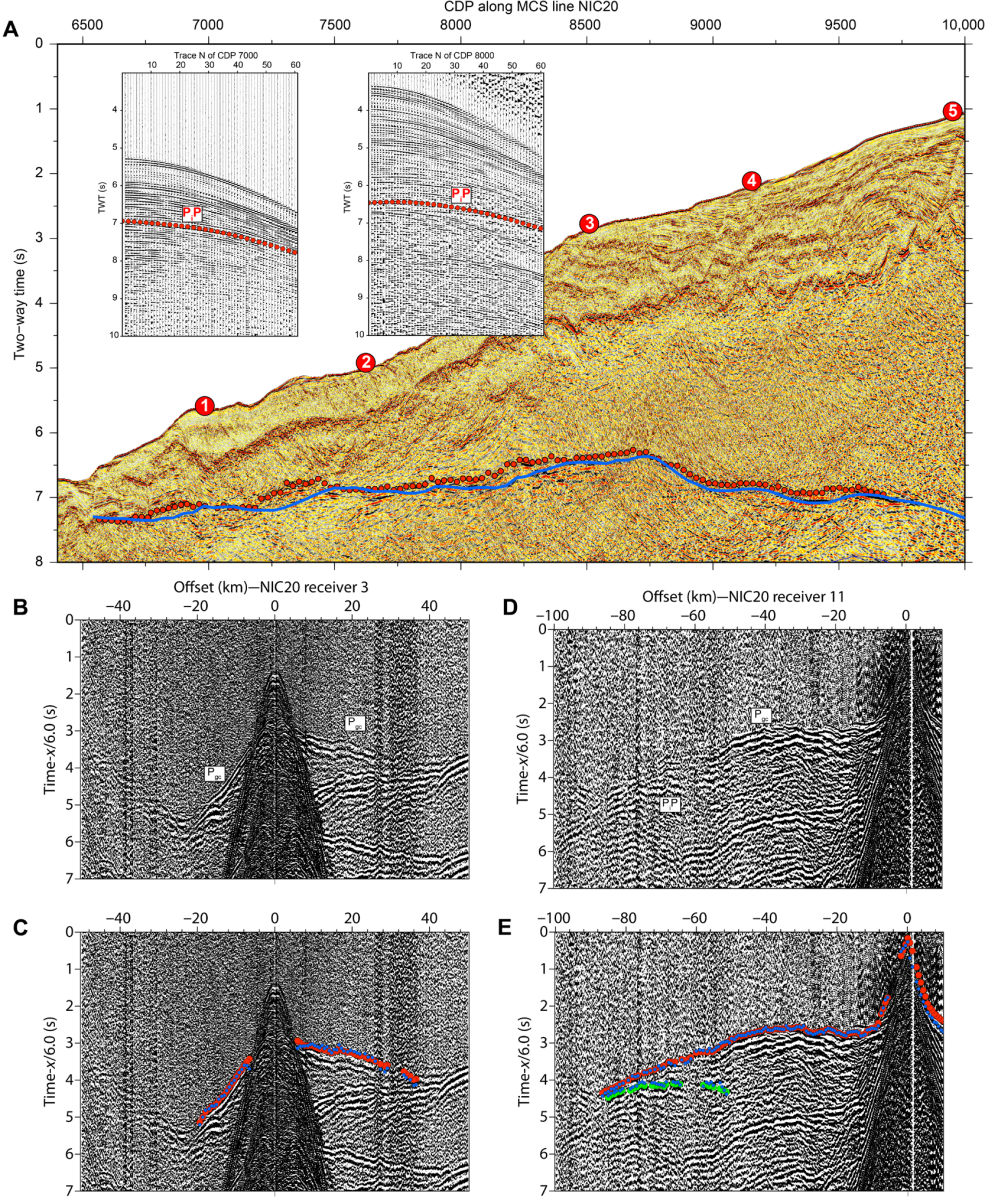
Examples of MCS and OBH seismic records. (**A**) MCS section along profile NIC20 near the trench showing OBH locations (red numbered circles) and the location of the interplate boundary reflector (red circles). The blue line is the inverted interplate boundary converted to two-way time (TWT) using *V*_P_ of NIC20 (Fig. 1B). The CDP spacing along the line is 12.5 m. The results show a good agreement between the observed and inverted interplate boundary (red circles and blue line, respectively). The insets display CDP gathers #7000 and #8000 with arrival times of P_i_P phases (red circles), corresponding to interplate reflections. The trace distance within CDP gathers is 100 m. (**B**) Record section of OBH-3 along profile NIC20. (**C**) Same record section as (B) showing picked (red circles) and synthetic (blue circles) P_gc_ travel times, corresponding to upper plate refractions. (**D**) Record section of OBH-11 along NIC20. (**E**) Same record section as (D) showing picked (red circles) and synthetic (blue circles) P_gc_ travel times and picked (green circles) and inverted (blue circles) P_i_P travel times.

The seismic dataset used here consists of coincident wide-angle reflection and refraction seismic (WAS) data and multichannel seismic (MCS) reflection data acquired along three trench-perpendicular profiles covering the rupture area of the Nicaragua 1992 earthquake (Fig. 1A). We performed a joint travel-time tomography of first arrivals and interplate reflections identified in both WAS and MCS record sections (Fig. 2 and figs. S1 to S6), following a statistical approach that is explained in Materials and Methods. This allowed us to retrieve the 2D *V*_P_ distribution and the geometry of the interplate boundary along each profile (Fig. 1B), together with the corresponding model parameter uncertainty (fig. S7). Overall, the three tomographic models show a similar *V*_P_ structure of the upper plate, with the strongest *V*_P_ changes concentrating in the near-trench zone, and a slightly variable interplate dip angle of about 12° to 15° (Fig. 1B).

**Fig. 3:**
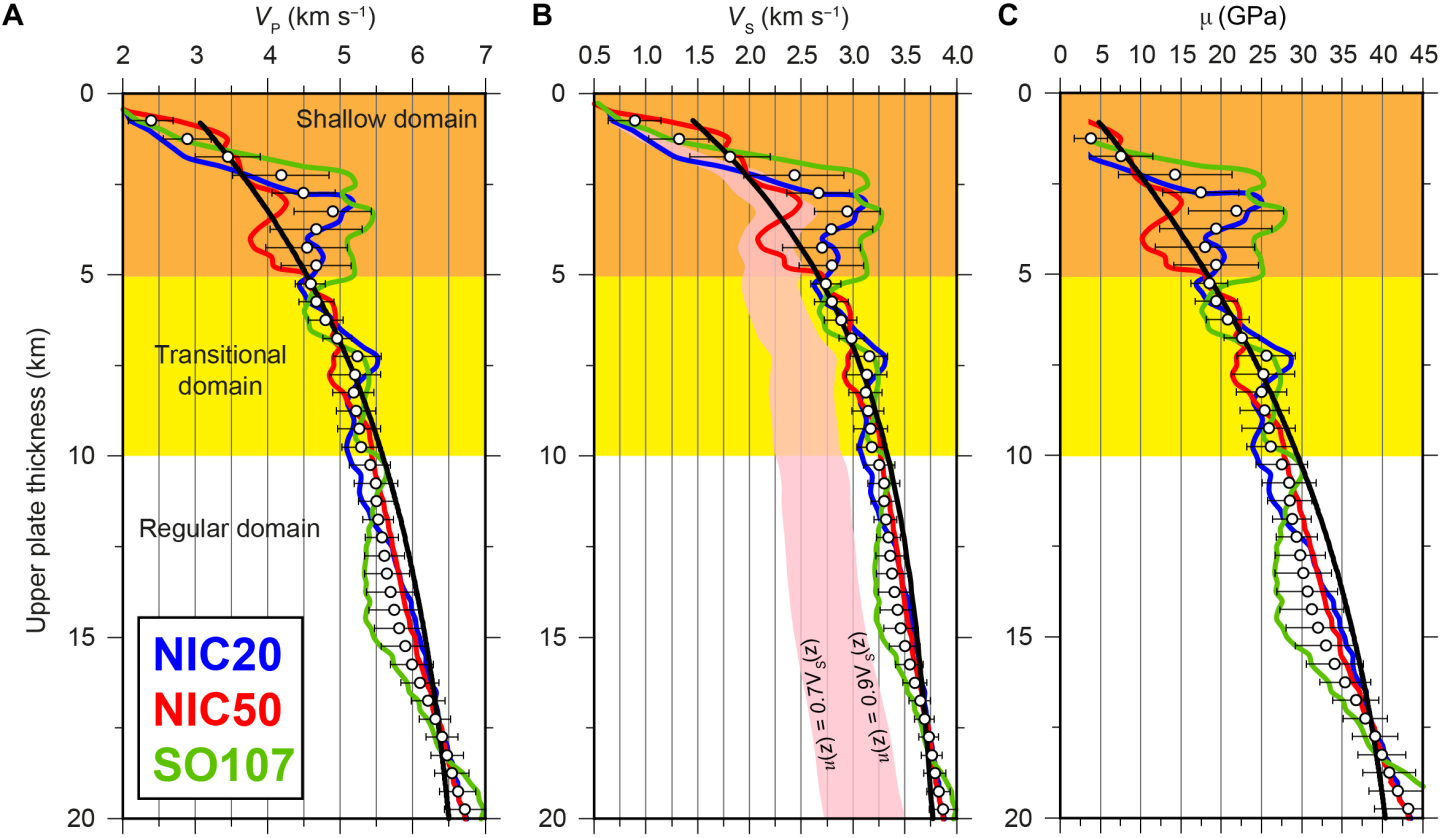
Elastic rock properties at the base of the upper plate. The values in the different panels are obtained from the 2D *V*_P_ models in Fig. 1B. (**A**) *V*_P_(*z*), (**B**) *V*_S_(*z*), and (**C**) μ(*z*). In all cases, *z* is the interplate boundary depth below the seafloor (bs). Blue lines correspond to NIC20, red lines correspond to NIC50, and green lines correspond to SO107. Black lines correspond to the worldwide average reported by Sallarès and Ranero (*19*). White dots represent the average of the three transects, and the error bar is 1 SD. The light pink area in (B) depicts the zone of possible rupture velocities (*u*) as a function of upper plate thickness, i.e., 0.7*V*_S_(*z*)*u* < 0.9*V*_S_(*z*). The depth extent of the shallow, transitional, and regular domains is taken from Sallarès and Ranero (*19*).

We then extracted *V*_P_(*z*), where *z* is the interplate boundary depth below the seafloor (bs) or upper plate thickness, just above the interplate boundary along the three profiles (Fig. 3A). *V*_P_(*z*) varies from about 2.0 km s^−1^ at 1 km bs to about 6.5 km s^−1^ at 20 km bs, but the variation is not uniform. The vertical *V*_P_ gradient is 0.13 s^−1^ between 5 and 20 km bs [within the segments classified as “regular” and “transitional” domains in the SR model (*19*) and in Fig. 3A], and sharply increases to 0.65 s^−1^ between 0.5 and 5 km bs [the “shallow” domain in (*19*) and in Fig. 3A]. The low *V*_P_ in this domain likely reflects the trenchward-increasing fracturing degree related to the pervasive upper plate faulting as compared to the deeper domains (*19*).

We then used *V*_P_(*z*) along the three profiles to calculate density (ρ), shear-wave velocity (*V*_S_), and rigidity ($\mu =\rho V_{S}^{2}$). For the conversion, we applied Brocher’s ρ(*V*_P_) and *V*_S_(*V*_P_) empirical relationships (*46*), which are based on experimental data at different conditions and combine existing relations for multiple rock types including those present in subduction zones. The obtained *V*_P_(*z*) is shown in Fig. 3A; ρ(*z*) is shown in fig. S9A; *V*_S_(*z*) and the limiting rupture propagation velocity *u*(*z*), which is 70 to 90% of *V*_S_ for dip-slip earthquakes, are shown in Fig. 3B; and μ(*z*) is shown in Fig. 3C. Similar to the *V*_P_ depth trend, the strongest variations in all these parameters concentrate in the shallow domain. *V*_S_(*z*) varies from about 0.5 to 2.75 km s^−1^ in the shallow domain and from 2.75 to 3.75 km s^−1^ in the transitional and regular domains, whereas μ(*z*) changes from about 5 GPa to 20 to 25 GPa in the shallow domain and from 20 to 25 GPa to 40 to 45 GPa in the transitional and regular domains. Therefore, the *V*_P_(*z*), *V*_S_(*z*), and μ(*z*) distributions follow the average trends obtained by polynomial regression of *V*_P_-derived properties of worldwide subduction zones (*19*) in the transitional and regular domains. The depth gradient is stronger in the model than in the average trend within the shallow domain, with values notably higher than the average close to the transitional domain and lower than the average near the toe of the wedge (Fig. 3). This discrepancy reflects the influence of the local changes in geology and rock fracturing degree because the frontal sediment prism ranges between 1 and 5 km in width and upper plate basaltic basement extends close to the trench axis (*31*).

To quantify the influence of these depth-varying elastic properties on the different source properties of the 1992 earthquake, we have first averaged *V*_P_(*z*) over the three profiles to obtain the mean depth trend and the corresponding *V*_P_ uncertainty (Fig. 3A), as is explained in Materials and Methods. This average trend captures the basic common attributes of *V*_P_(*z*) along the three lines and filters out the local lateral heterogeneity of the shallow domain. We have then derived the rest of properties (i.e., *V*_S_, *u*, ρ, and μ) and have finally extended them laterally to obtain the corresponding 2D maps over the earthquake’s rupture zone (fig. S10).

### Moment release, slip, duration, and stress drop of the 1992 tsunami earthquake

We have performed a finite fault inversion of the moment release and slip distribution that conforms to local information concerning the orientation of the subduction trench (312°) and the average dip angle of subduction issued from the seismic data (15°), assuming that plate convergence is perpendicular to the trench (fig. S11A). We impose layered 1D velocity and rigidity profiles that follow the depth distribution of elastic properties extracted from the tomography models (fig. S11G). The total seismic moment that we obtain for this event is 3.36 × 10^20^ N·m (*M*_W_7.62), and the total duration is near 140 s (fig. S11B), for an explored rupture area that is 286 km long and 71.5 km wide. Additional details on the data used and the inversion method are provided in Materials and Methods.

**Fig. 4:**
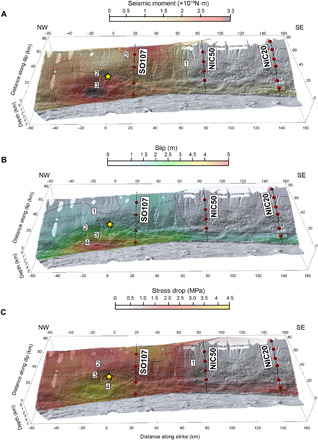
Released moment, slip, and stress drop on the 1992 Nicaragua earthquake. Front view of the Nicaraguan margin showing the calculated distribution of source properties throughout the rupture area of the 1992 Nicaragua tsunami earthquake. (**A**) Moment release in N·m according to the color scale. (**B**) Co-seismic slip in meters according to the color scale. (**C**) Stress drop in MPa according to the color scale. In all cases, the yellow star indicates the epicenter location. Black lines are the seismic profiles, and red circles are OBHs.

The moment release distribution displays two main patches, one located within the shallow domain, trenchward from the epicenter, and the second one some 20 km landward toward southeast (SE) (Fig. 4A and fig. S12A). There is also significant moment release up to 80 km northwest (NW) from the epicenter and 60 km SE from it, although in the shallow domain of this SE part there is substantial moment release up to 160 km from the epicenter. The slip distribution that results from the depth-dependent rigidity profile displays a large slip patch in the shallowest part of the megathrust to 50 to 60 km to either side of the epicenter (fig. S12B and Fig. 4B). The average slip in this patch is 4 m, with peak values exceeding 5 m. There is also significant slip of 1 to 2 m at both sides of the epicenter and all along the near-trench part of the fault. We performed other finite slip inversion tests exploring narrower rupture zones that provide similar moment release and slip distributions, with overall larger slip reaching 8 m in the main patch and 2 to 3 m within the shallow domain.

Our model of physical properties also sets physical limitations to the moment release time in the different parts of the fault. Shear stresses must accumulate at the crack tip for spontaneous rupture propagation, so the *u* field in Fig. 3B should limit the earliest possible onset release time at each point of the fault. The main observation to constrain the average rupture velocity of this event is the total duration (~140 s). This long duration is mainly caused by a rupture propagating laterally up to 160 km toward the SE along the shallowest portion of the megathrust (Fig. 4B). This corresponds to a slow average rupture propagation velocity of 1.1 to 1.2 km s^−1^ toward the SE. For comparison, fig. S9C shows the average *V*_S_ as a function of the distance to the trench, which is 1.5 km s^−1^ from 0 to 5 km, with *u* ranging between 1.05 and 1.35 km s^−1^, and it is 2.5 km s^−1^ from 5 to 10 km, with *u* between 1.75 and 2.25 km s^−1^. The anomalously long rupture duration and slow propagation velocity are thus compatible with a rupture propagating laterally along the shallowest tip of the megathrust.

Another parameter of seismological interest is the static stress drop (Δσ), which corresponds to the decay of shear stress during the earthquake and influences important aspects of the seismic rupture. As in the case of slip, it can vary significantly over the rupture area, but in most cases, we can only estimate its spatial average, as

$$\bar{∆\sigma }\approx b\bar{\mu }\bar{\delta }L^{-1}$$ (1)

where $\bar{\mu }$ is the average rock rigidity, $\bar{\delta }$ is the average slip; *L* is a characteristic rupture dimension, often approximated as *S*^−1/2^; and *b* is a nondimensional geometric factor of order unit that depends on fault geometry and on the elastic moduli (*47*). This approximation is considered to be valid unless stress variations along the fault are very large.

Given that we mapped the depth distribution of rigidity (figs. S9E and S10D), the elastic moduli to calculate *b*, and slip (Fig. 4B and fig. S12B) throughout the rupture area, we can apply Eq. 1 to each individual subfault segment to derive a stress drop distribution that is consistent with the seismological data and the elastic rock property distribution (Fig. 4C and fig. S12C) (see Materials and Methods for details). In our model, the maximum values of stress drop occur at the patch of largest moment release located trenchward from the epicenter (4 to 5 MPa) and 20 km NE from it (3 to 4 MPa) with smaller values elsewhere across the rupture area. The slip-weighted average over the entire rupture area is $\bar{\text{∆}\sigma }$ = 2.2 MPa, within the global average of subduction megathrust events (*48*).

## DISCUSSION

### Comparison with previous models and field observations

Previous slip models of the Nicaragua 1992 tsunami earthquake differ substantially in the extent and spatial distribution of slip because of limitations of the data used and intrinsic trade-offs between slip and other source parameters (*37*–*45*). Most models assume a uniform slip concentrating in a 150- to 280-km-long band located trenchward from the epicenter and try to match seismological and/or tsunami data with different combinations of rupture zone width (*w*), slip, and rigidity. Body wave analysis assuming μ = 30 GPa suggests a value of $\bar{\delta }$ ranging from 0.5 m for *w* = 100 km (*42*) to 1.4 m for *w* = 50 km (*44*). Tsunami run-up data favor the latter option but with significantly lower rigidity. A wide rupture zone with *w* = 100 km, μ = 30 GPa, and $\bar{\delta }$=3.75 m fits the wave heights but overestimates *M_0_* by one order of magnitude (*37*), whereas a narrower fault with *w* = 40 km, μ = 10 GPa, and $\bar{\delta }$=3 m explains better both observations (*36*, *39*). Some variable slip models also support a narrow, low-rigidity rupture zone but a patchy slip distribution. Piatanesi *et al.* (*40*) fitted tsunami run-up with μ = 10 GPa and a fault composed of five 50 km × 50 km sectors, obtaining a preferred solution with 3.5 to 4.5 m of slip near the NW and SE limits of the rupture area and 1 to 2 m elsewhere. A comparable slip distribution was obtained using the heterogeneous moment release model in Fig. 1A and μ = 22 GPa (*35*). The seismic and tsunami models were reconciled, combining a spatially variable moment release distribution (*33*) with a depth-varying rigidity increasing from 3.6 to 30 GPa (*45*). Although they did not provide slip values, the comparatively lower rigidity of the shallow megathrust implies that shallow slip must be larger than the 3.5 to 4.5 m estimated with the spatially variable moment release (*33*) to match the observations. In contrast, finite fault solutions obtained using a variable rigidity profile extracted from the Crust 1.0 model favor moderate slip with maximum values of 1.2 to 1.5 m across a wider rupture zone (*49*).

Our model supports the occurrence of large shallow slip reaching 5 m in the near-trench low-rigidity (3 to 10 GPa) area (Fig. 3C and fig. S10D) but with a more continuous slip distribution along the shallow domain than in previous variable slip models (Fig. 4B) (*33*). Shallow slip may further increase to 8 m if the rupture zone width is restricted to 40 to 50 km. Even larger near-trench slip has been estimated for other tsunami earthquakes such as the *M*_S_7.2 1896 in Sanriku (9.5 to 10 m) (*50*), the *M*_S_7.4 1946 in the Aleutians (10 to 11 m) (*51*), or the *M*_S_7.1 2010 in Mentawai (9 to 10 m) (*52*), assuming low rigidity in all cases. In contrast to the site-specific models that attribute large slip and seafloor displacement to the presence of local features enhancing normal deformation (*12*–*14*), or to particular conditions reducing fault friction (*7*–*9*), in our model, the large shallow slip is a natural consequence of a rupture concentrating in the low-rigidity near-trench rocks. In other words, in this case, site-specific factors do not appear to be necessary to produce the inferred large near-trench seafloor deformation. The key difference between all the previous slip models for Nicaragua or any other tsunami earthquake and ours is that, rather than assuming or imposing any ad hoc constraint on rock properties above the megathrust, we extract them from local, controlled-source seismic tomography models.

Aside from providing a slip distribution that is consistent with the seismic tomography models, the obtained rock properties throughout the rupture area allow us to explain other observations and to answer additional open questions. The different source models of the Nicaragua earthquake confirm that it had a long duration of 100 to 150 s so that the rupture propagation was slow, of 1.0 to 2.2 km s^−1^ on average (*28*, *32*, *42*, *43*). As stated above, this range of values agrees with the estimated limiting propagation velocity of the shallow domain (i.e., within 10 to 20 km from the trench), which is 1.0 to 2.3 km s^−1^, assuming *u =* 0.7*V_S_* (fig. S10C).

The static stress drop influences important source properties such as the moment-rate spectrum, but it is difficult to estimate, as it has large trade-offs with other parameters such as *V*_S_, μ, or δ. In the case of the Nicaragua 1992 event, stress drop estimations range from values as low as 0.08 to 0.26 MPa (*38*, *42*), intermediate ones of about 0.78 to 1 MPa (*44*, *49*), to values of up to 3 to 7 MPa (*33*). In all these cases, strong assumptions on the values of the elastic rock properties were made to fit the observation. Our spatially variable and elastic property–consistent stress drop model supports intermediate values of 2 to 4 MPa, but with an irregular distribution with maximum values of 4 to 5 MPa concentrating in the near-trench patch of largest slip (Fig. 4B). As stated above, these average Δσ values are close to the global average in subduction zones, of 2 to 3 MPa (*48*).

In previous studies, the main argument put forward to justify low Δσ values is the high-frequency depletion of the moment spectrum (*38*, *49*). The energy decay occurs after the corner frequency, *f_c_*, which is expressed as

$$f_{c}=cV_{s}{\left(\frac{∆\sigma }{M_{0}}\right)}^{1/3}$$ (2)

where *c* is a dimensionless constant.

**Fig. 5:**
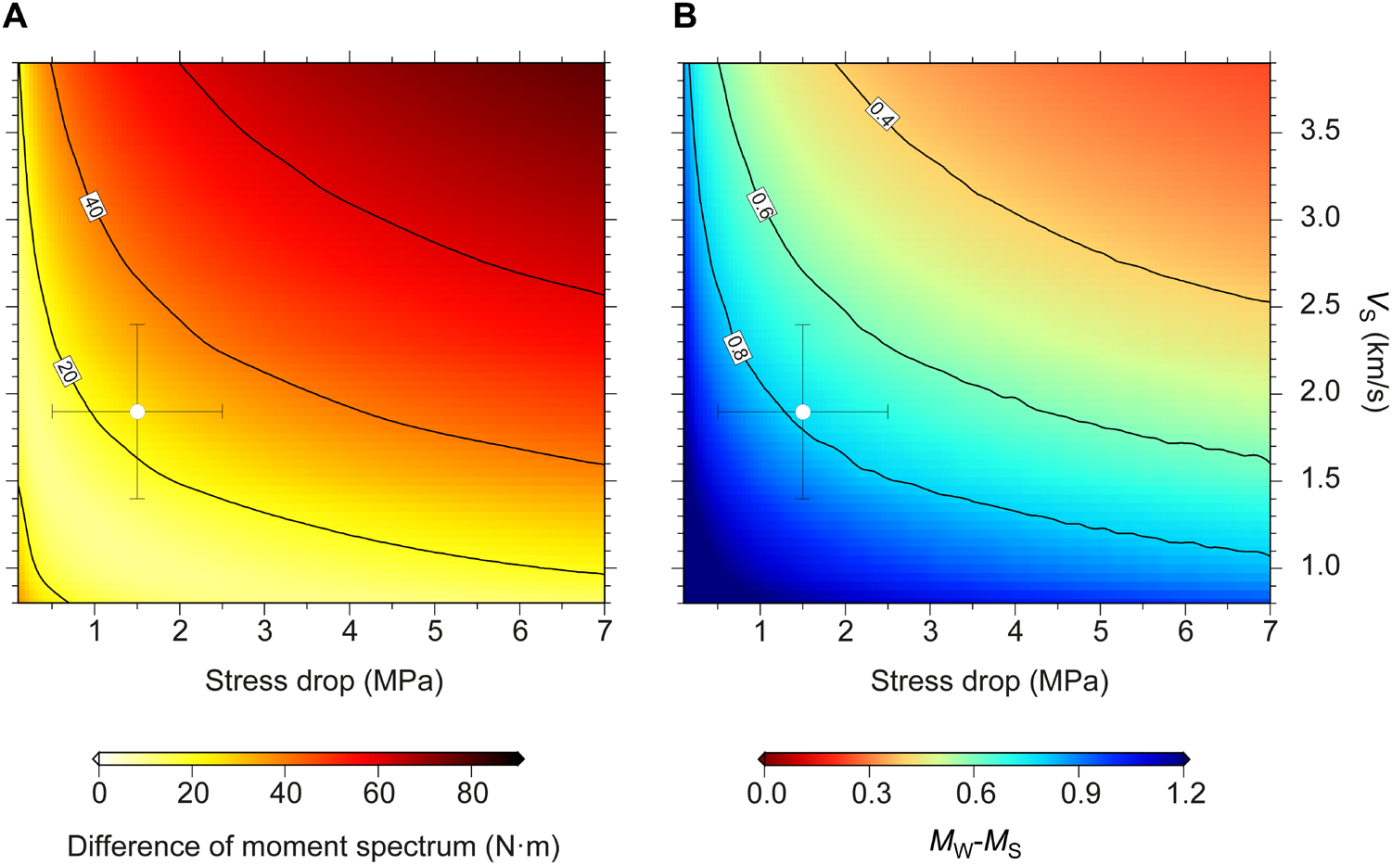
Moment spectrum and magnitude discrepancy residuals. (**A**) Root mean square residual between the observed moment-rate spectrum of the Nicaragua 1992 tsunami earthquake (black line in Fig. 6) and that calculated for different combinations of *V*_S_ and Δσ. Units are N·m·10^−18^, and the color code follows the corresponding scale. (**B**) Calculated difference between *M*_W_ and *M*_S_, as a function of depth, for an earthquake of *M*_W_ = 7.7 such as the 1992 Nicaragua event. *M*_W_ and *M*_S_ are estimated using the computed moment amplitude $\left(\overset{\cdot }{M}\right)$ at periods of 250 and 20 s, respectively, with $\overset{\cdot }{M}(f)=\frac{M_{0}f_{c}^{n}}{f^{n}+f_{c}^{n}}$, taking *f_c_*(*z*) in Eq. 2 and *V*_S_(*z*) in Fig. 3B. The white circle in (A) and (B) indicates the average *V*_S_ and Δσ values of the near-trench segment (within 10 km from the trench) and their SDs (*V*_S_ = 1.90 ± 0.40 km s^−1^, Δσ = 1.85 ± 0.50 MPa). This is the fault segment where most slip concentrates (Fig. 4A).

**Fig. 6:**
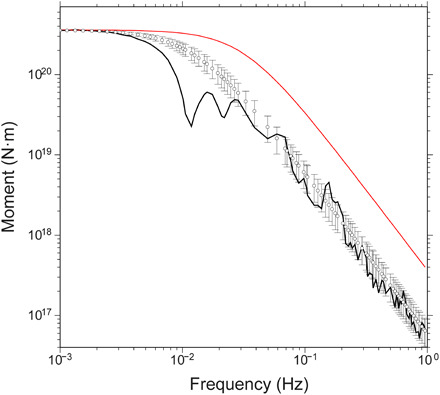
Observed versus calculated moment spectrum. The black line shows the observed moment spectrum of the Nicaragua 1992 earthquake (*49*). White dots correspond to the average moment-rate spectrum of models estimated using combinations of *V*_S_ within 1.90 ± 0.50 km s^−1^ and Δσ within 1.85 ± 0.50 MPa. Error bars are 1 SD. The red line is the reference spectrum obtained with *V*_S_ = 3.75 km/s and Δσ = 3 MPa.

Therefore, for a given *M*_0_ and *V*_S_, *f_c_* is proportional to ∆σ^1/3^, while the dependence on *V*_S_ is linear, so the effects of moderate changes in *V*_S_ can be stronger than those in Δσ. As it is shown in Fig. 5, a range of Δσ-*V*_S_ combinations could explain the moment-rate spectrum of the 1992 earthquake, but most of them are not compatible with the inferred elastic properties throughout the earthquake’s rupture zone. In their work, Ye *et al.* (*38*) estimated Δσ = 0.08 MPa, assuming *V*_S_ = 3.75 km s^−1^, which is the velocity for undamaged crystalline rock that we obtain in the regular domain (Fig. 3B), where there is almost no slip (fig. S12B). However, the moment-rate spectrum can also be fitted with higher average values of stress drop if *V*_S_ is lower (Fig. 5A). In particular, we show that it can be explained with *V*_S_ = 1.90 ± 0.4 km s^−1^ and Δσ = 1.85 ± 0.5 MPa (Fig. 6), which are the average values in the near-trench zone (fig. S9), where the largest slip concentrates (Fig. 4B). Because *M*_S_ is calculated at higher frequencies than *M*_W_ (at 50 and 4 mHz, respectively), the high-frequency depletion caused by the low *V*_S_ increases the *M*_W_*-M*_S_ difference. As it is shown in Fig. 5B, the average near-trench *V*_S_ and Δσ values referred above can also account for the *M*_W_*-M*_S_ difference of up to 0.7 estimated for the Nicaragua 1992 earthquake, which is, in turn, similar to the differences found in other tsunami earthquakes (*51*, *52*).

In summary, the mapped distribution of elastic rock properties across the rupture zone of the 1992 Nicaragua tsunami earthquake reproduces slip patterns and duration times that are consistent with observations from seismological and tsunami data. In addition, it provides field data–based constraints to estimate a stress drop distribution that reproduces, in turn, the observed moment spectrum and the *M*_W_*-M*_S_ difference. Although local geology and tectonics, changes in frictional conditions, or ancillary sources may play a significant role in shallow rupture, seafloor deformation, and tsunamigenesis, the influence of these site-specific factors should be analyzed without ignoring the underlying and universal effect of the depth-varying upper plate rock elasticity. Obtaining accurate information on the distribution of elastic properties of the compliant upper plate rocks undergoing deformation during the earthquake, and incorporating it into dynamic rupture models, is therefore key to properly characterizing rupture behavior and the resulting seafloor deformation. This is a key parameter to properly estimating tsunami wave heights to improve, in turn, predictive tsunami forecasting and hazard assessment. Inconsistencies found in the slip estimated from different types of data (e.g., seismological, geodetic, or tsunami) for the Nicaragua 1992 event or for recent large and giant earthquakes (*53*, *54*) could well be due to inaccurate assumptions or oversimplifications on the estimation of the elastic property distribution across the rupture zone. Local seismic surveys providing *P*-wave and *S*-wave seismic velocity as well as the geometry of the interplate boundary in hazardous areas appear as a key element to retrieve the necessary information to reproduce earthquake rupture scenarios under realistic conditions. Ignoring these variations in rock properties may induce substantial biases in the estimated source properties, particularly for shallow ruptures, so that the tsunamigenic potential of the associated tectonic structures can be severely underestimated.

In addition, our results show that the long duration, high-frequency depletion, and *M*_W_*-M*_S_ discrepancy are all inherent properties of ruptures concentrating in the low-rigidity rocks that are present at the near-trench megathrust zone (Figs. 5 and 6), so these attributes are strong indicators of enhanced tsunami hazard for earthquakes of similar magnitude and focal depth. For instance, the 2016 Ecuador earthquake of *M*_W_7.8 had slightly larger seismic moment than the Nicaragua 1992 one and a focal depth of 18 to 20 km (*55*), but as it ruptured mainly downdip, it displayed no high-frequency deficit or anomalously long duration (*56*) and did not cause a tsunami. We propose that this type of information should be taken into consideration to improve hazard assessment in tsunami early warning systems.

## MATERIALS AND METHODS

### Controlled-source seismic dataset and travel-time picking

Wide-angle seismic (WAS) data along lines NIC20 and NIC50 were acquired in 2000 during U.S. *R/V Maurice Ewing* cruise EW00-05 (*57*), while WAS data along SO107 were acquired in 1996 during the SO107 cruise of the German *R/V Sonne* (*58*). The data were recorded by 11 ocean bottom hydrophones (OBHs) along NIC20, 10 OBHs along NIC50, and 7 OBHs along SO107/NIC80. Land stations were also deployed along each line to record offshore shots. We used four of those in the modeling of NIC50 (Fig. 1A). We relocated the OBH using near-offset water wave first arrival times, and a 7- to 15-Hz band-pass filtering was applied before travel-time picking.

Coincident MCS data along NIC20, NIC50, and SO107 (originally, NIC80) were also acquired during EW00-05 survey, using a 6-km-long streamer and an airgun array of 112 liters as seismic source (*57*). We used processed field and stacked data from the MCS sections (*30*, *59*) to pick arrival times of *P*-wave reflections at the shallow interplate boundary (P_i_P in figs. S1 to S3). In particular, we picked travel times in common depth point (CDP) gathers and used the corresponding stack section for quality control and travel-time crosschecking. We picked every 25th CDP, which, for a CDP spacing of 12.5 m, corresponds to a picking distance of ~300 m. This is twice the lateral spacing of the tomographic grid (150 m). Additional testing of lower grid size and CDP spacing for picking did not improve the tomography results. We used WAS records to pick travel times of refracted *P* wave through the upper plate (P_gc_ in Fig. 2 and figs. S4 to S6) and P_i_P travel times of the deep section of the interplate boundary (P_i_P in Fig. 2D and figs. S4 to S6), where we could not pick interplate reflection arrivals at CDP gathers because of the low signal-to-noise ratio.

In total, we picked 1229 P_i_P and 5549 P_gc_ travel times along NIC20, 1058 P_i_P and 4537 P_gc_ travel times along NIC50, and 1925 P_i_P and 2354 P_gc_ travel times along SO107. Travel-time picking error ranges between ±20 and 40 ms for MCS P_i_P travel times, ±30 and 60 ms for P_gc_ WAS travel times, and ±60 and 90 ms for P_i_P WAS travel times. These values are estimated on the basis of the amplitude ratio of 250-ms-wide windows recorded just before and after the picks.

### Joint refraction and reflection travel-time tomography and statistical uncertainty analysis

There are previously existing 2D *V*_P_ models of the upper plate and the geometry of the interplate boundary along NIC20 (*59*) and SO107/NIC80 [NIC1 in (*58*)] profiles, but not along NIC50. The NIC20 model was obtained by joint refraction and reflection travel-time tomography, whereas SO107/NIC80 was constructed by forward modeling. In the two cases, the published *V*_P_ models were obtained using WAS travel times alone. To make the three *V*_P_ models directly comparable, we have modeled these two profiles again, together with NIC50, using identical model parameterization, regularization constraints, inversion strategy, and uncertainty analysis.

In contrast with the two previous studies, here, we jointly inverted travel times from both WAS and MCS records along the three profiles. For this, we used a modified version of the joint reflection and refraction travel-time tomography code tomo2D (*60*) that can also handle MCS data (*61*). While WAS data provide travel-time information at long offsets (up to ~100 km), MCS data are restricted to the streamer length (a maximum offset of 6 km in this case). However, the denser spatial sampling of MCS data translates into a much larger number of reflected travel times than in WAS data. Therefore, combining MCS and WAS travel times yields a better ray coverage of shallow interfaces and, thus, lower velocity and reflector geometry uncertainty.

We inverted travel times following a statistical Monte-Carlo approach that provides uncertainty estimates of model parameters along each profile (i.e., *V*_P_ and reflector geometry) (fig. S7). We performed 100 different inversions (Monte Carlo realizations) for each profile using different starting *V*_P_ models and initial interplate reflectors, combined with travel-time data sets that are perturbed with random Gaussian noise of similar amplitude as the picking error. The travel-time noise includes common receiver, common phase, and individual travel-time picking errors that sum up to ±80 ms. Starting models are generated by randomly varying by ±10% the *V*_P_ of a reference model that consists of a vertical velocity gradient with *V*_P_ increasing from 1.8 km s^−1^ at the top to 8.2 km s^−1^ at the bottom. Each initial interplate reflector has a constant dip angle that varies between 8° and 15°.

Each starting model is parameterized as a regular grid hanging from the seafloor, with a horizontal node spacing of 150 m and a variable vertical spacing increasing from 50 m at the top to 500 m at the bottom of the model, which is located at a depth of 50 km. The lateral node spacing for the interplate reflector is 150 m. Regularization parameters for the *V*_P_ field are defined by imposing horizontal and vertical correlation lengths (CLs) that control the smoothing of the inverted model. In this study, vertical *V*_P_ CL increases from 1 km at the top of the model to 2 km at the bottom, whereas the horizontal *V*_P_ CL increases from 1 km at the top of the model to 5 km at the bottom. The CL for the interplate reflector is 4 km.

The results of each realization were obtained after 10 iterations. All realizations converged satisfactorily from initial root mean square values of travel-time residuals of 0.5 to 1.0 s to final values of 50 to 60 ms (fig. S8). For each profile, we have computed the average 2D *V*_P_ model and the average geometry of the interplate boundary for the 100 realizations (Fig. 1B and fig. S7, D, H, and L) and their SDs (fig. S7, B, F, and J), which are a proxy of the model parameter uncertainty (*60*).

Overall, *V*_P_ uncertainty rarely exceeds 0.2 km/s (fig. S7, B, F, and J). Larger *V*_P_ uncertainties of 0.2 to 0.4 km/s are found in ill-constrained regions because of poor ray coverage, such as the bottom of NIC50 or localized segments along SO107 (fig. S7, F and J). The latter profile has half the amount of OBHs than the rest of the lines over the same profile distance and, thus, a lower number of P_gc_ travel times and a poorer ray coverage of the upper plate (fig. S7J). Interplate depth uncertainty is lower than 50 m for the shallow region, where it is covered by MCS P_i_P travel times, and increases to 0.5 to 1.0 km in the deeper region, where it is just covered by a limited number of P_i_P travel times from WAS data (fig. S7F).

### Finite fault inversion with realistic depth-varying elastic properties

To invert this earthquake, we have taken into consideration available tectonic information, mainly from multibeam bathymetry and from the seismic data used in this work, which allowed us to define the location of the trench, plate convergence direction, and geometry of the subduction interface. To be consistent with this information, we slightly changed the plane from the GCMT (Global Centroid Moment Tensor) solution, which was strike 303°, dip 12°, and rake 91° to 312°, 15°, and 91°, respectively (fig. S11A). We then constrained the geometry of the fault plane following the trench in the strike direction and the subduction interface along dip. We used P waveform data recorded at 15 teleseismic broadband stations (fig. S11C), SH waveforms recorded at 6 broadband stations (fig. S11D), and long period surface waves recorded at 19 stations (fig. S11, E and F). The stations were selected on the basis of data quality and azimuthal distribution. Waveforms were first converted to displacement by removing the instrument response and then used to constrain the slip history using a finite fault inverse algorithm (*62*, *63*). We started the inversion using a hypocentral location matching the initial solution provided by the National Earthquake Information Center (NEIC), located at 11.5°N, 87.6°W with a depth of 9.5 km. Note that this depth value is consistent with the interplate depth retrieved from the tomography models at the epicentral location (fig. S10E). We used local reference velocity and rigidity models corresponding to 1D layered profiles extracted from the controlled-source tomography results of this work (fig. S11G).

### Estimation of stress drop distribution

The stress drop distribution (fig. S12C) was obtained applying Eq. 1 to each cell with the slip values in fig. S12B, *L* = 10 km, $b=\frac{4}{\pi }\frac{\left(M-\mu \right)}{M}$, where Μ is the *P*-wave modulus at the cell’s location and μ is the resampled rigidity distribution. For display purposes, we smoothed the slip and stress drop models in fig. S12 (B and C) by applying a 2D Delaunay triangulation filter and superimposed them on the multibeam bathymetry map. The resulting maps are displayed in Fig. 4 (B and C, respectively).

## SUPPLEMENTARY MATERIALS

Supplementary material for this article is available at http://advances.sciencemag.org/cgi/content/full/7/32/eabg8659/DC1
